# Supplementary material for: A high-resolution mRNA expression time course of embryonic development in zebrafish
Source: eLife. 2017 Nov 16;6:e30860. doi: 10.7554/eLife.30860 (PMC5690287; doi:10.7554/eLife.30860)
Supplement: Supplementary file 6. [file elife-30860-supp6.zip › biolayout-clusters-files/Cluster234-genes.html]

Cluster234


# Cluster234: Genes

| Ensembl ID | Gene Name | Chr | Start | End | Biotype |
| --- | --- | --- | --- | --- | --- |
| ENSDARG00000058853 | SASH1 (1 of many) | 17 | 6830274 | 6915597 | protein\_coding |
| ENSDARG00000004311 | ldlrap1a | 19 | 29750548 | 29776559 | protein\_coding |
| ENSDARG00000028559 | march5l | 10 | 42672515 | 42691393 | protein\_coding |
| ENSDARG00000004470 | prkcsh | 3 | 48810369 | 48842955 | protein\_coding |
| ENSDARG00000044601 | rtn4a | 6 | 6073843 | 6101282 | protein\_coding |
| ENSDARG00000051857 | tes | 25 | 18378629 | 18400010 | protein\_coding |
